# The mycelium of the *Trametes versicolor* (Turkey tail) mushroom and its fermented substrate each show potent and complementary immune activating properties in vitro

**DOI:** 10.1186/s12906-019-2681-7

**Published:** 2019-12-02

**Authors:** Kathleen F. Benson, Paul Stamets, Renee Davis, Regan Nally, Alex Taylor, Sonya Slater, Gitte S. Jensen

**Affiliations:** 1 NIS Labs, 1437 Esplanade, Klamath Falls, Oregon, 97601 USA; 2 Fungi Perfecti, Postal Box 7634, Olympia, Washington, 98507 USA

**Keywords:** Anti-inflammatory, Immune modulation, CD69, Cytokines

## Abstract

**Background:**

The medicinal mushroom *Trametes versicolor* (Tv, Turkey Tail) is often prepared for consumption as a powder from the fungal mycelium and the fermented substrate on which it grew. The goal for this study was to evaluate the immune-modulating properties of the mycelium versus the fermented substrate, to document whether an important part of the immune-activating effects resides in the metabolically fermented substrate.

**Methods:**

Tv mycelium was cultured on rice flour. The mycelium and the fermented substrate were mechanically separated, dried, and milled. The initial substrate served as a control. Aqueous fractions were extracted and passed through 0.22-μm filters. The remaining solids were passed through homogenization spin columns without filtration. The aqueous and solid fractions of the initial substrate (IS), the fermented substrate (FS), and the *Trametes versicolor* mycelium (TvM) were tested for immune-activating and modulating activities on human peripheral blood mononuclear cell cultures, to examine expression of the CD69 activation marker on lymphocytes versus monocytes, and on the T, NKT, and NK lymphocyte subsets. Culture supernatants were tested for cytokines using Luminex arrays.

**Results:**

Both aqueous and solid fractions of TvM triggered robust induction of CD69 on lymphocytes and monocytes, whereas FS only triggered minor induction of CD69, and IS had no activating effect. The aqueous extract of TvM had stronger activating effects than the solid fraction. In contrast, the solid fraction of IS triggered a reduction in CD69, below levels on untreated cells.

Both aqueous and solid fractions of FS triggered large and dose-dependent increases in immune-activating pro-inflammatory cytokines (IL-2, IL-6), anti-inflammatory cytokines Interleukin-1 receptor antagonist (IL-1ra) and Interleukin-10 (IL-10), anti-viral cytokines interferon-gamma (IFN-γ) and Macrophage Inflammatory Protein-alpha (MIP-1α), as well as Granulocyte-Colony Stimulating Factor (G-CSF) and Interleukin-8 (IL-8). TvM triggered more modest cytokine increases. The aqueous extract of IS showed no effects, whereas the solid fraction showed modest effects on induction of cytokines and growth factors.

**Conclusion:**

The results demonstrated that the immune-activating bioactivity of a mycelial-based medicinal mushroom preparation is a combination of the mycelium itself (including insoluble beta-glucans, and also water-soluble components), and the highly bioactive, metabolically fermented substrate, not present in the initial substrate.

## Background

Medicinal mushrooms describe a category of edible members of the kingdom Fungi, traditionally associated with health-supporting properties. They have been used for centuries to treat an array of ailments, particularly in traditional Asian medicine and Eastern European traditions. They are well regarded for supporting longevity, treating infectious disease and cancer, and promoting overall well-being [1, 2]. Contemporary research has mainly focused on the broad immune activity of mushrooms. Several preclinical findings suggest that mushrooms may specifically support NK cell upregulation, [2–5] enhancement of T-cell and NK cell cytotoxicity [6], and the induction of immune-regulating cytokines such as TNF-α, IL-2, IFN-y, and IL-10 [7–10]. As a category, medicinal mushrooms stimulate host defense and immunity due to the complex and varying polysaccharides; some well-studied examples include (1,3;1,6)-β-glucans, proteoglycans, heteroglucans, that comprise the chitin-based fungal cell wall [11–13]. Mushrooms are also the source of other pharmacologically relevant compounds, such as proteins like Ling zhi-8 in *Ganoderma lucidum* [14] and lectins in several species [15], triterpenes [16, 17], phenols [18], and sterols [16]. While medicinal mushrooms generally confer broad immune activity, individual species often possess unique immunological properties. *Trametes versicolor* (Tv), commonly known as Turkey tail and previously named *Coriolus versicolor*, is known to enhance innate and adaptive immune responses [19, 20]. Recent clinical research involving consumption of Tv mycelium on rice substrate by Standish and colleagues [21] suggests NK cell induction in women with breast cancer. Other researchers cite antitumor effects [4, 22, 23], but this is generally considered to be a result of its underlying immunologic activity [24]. Tv contains precursors to the proteoglycans polysaccharide peptide (PSP) and polysaccharide-K (Krestin, PSK), the latter of which is frequently prescribed to gastric cancer patients in Japan [25]. The constituents responsible for these immunological effects are believed to be the polysaccharides. However, recent research suggests that the lipid fraction of PSK isolated from Tv is instrumental to its TLR-2 induction activity [26].

**Fig. 1:**
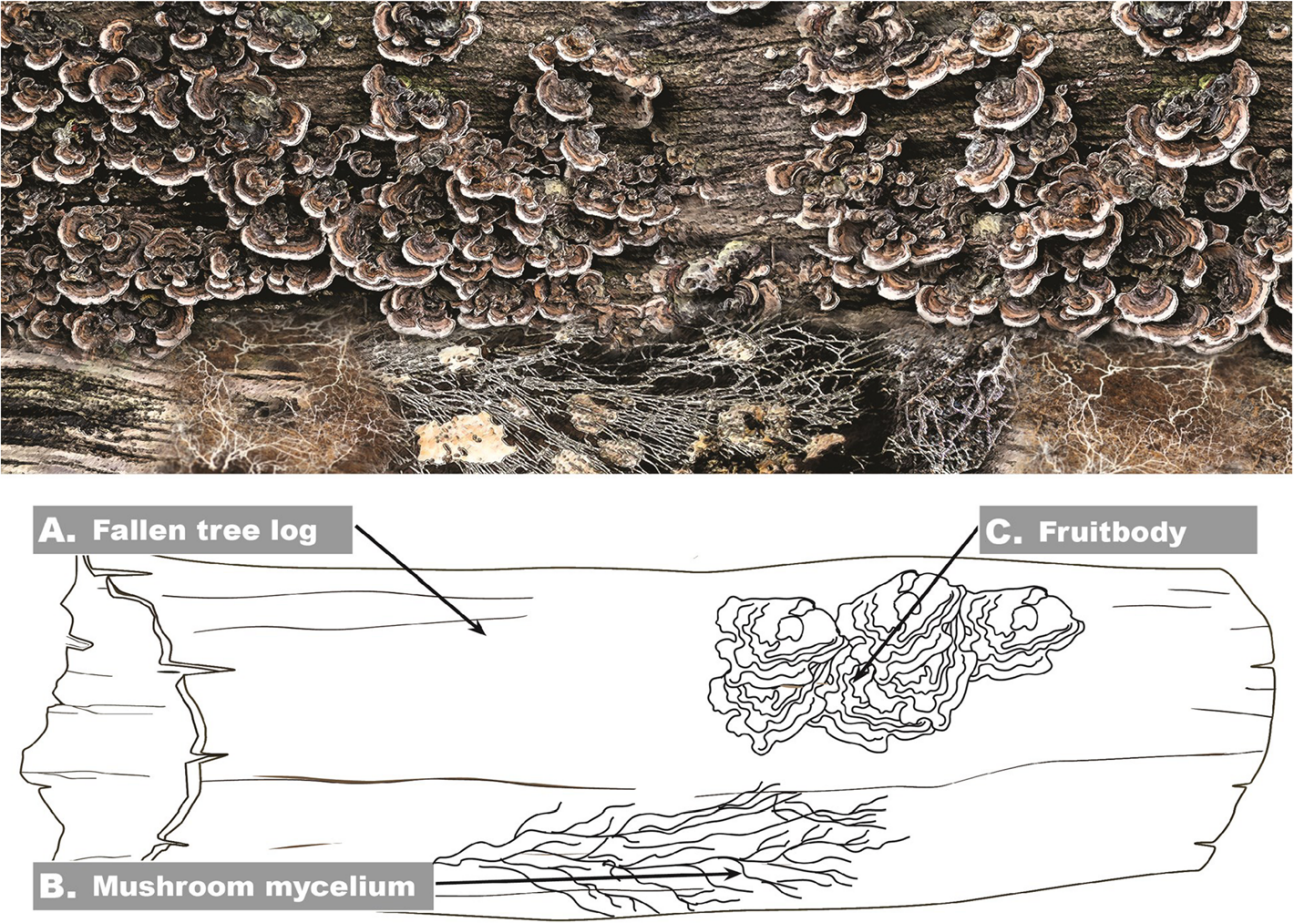
Decomposition of a fallen tree log by Trametes versicolor (Tv). **a** Fallen tree log, presenting fresh organic plant matter. **b** Tv mycelium is growing inside the log, decomposing the plant biomass by fermentation, in a highly dynamic exchange of solubilized nutrients from the tree log, resulting from secreted fungal enzymes, combined with anti-microbial defense compounds to protect the mycelial territory. **c** Fruitbodies serve to spread the spores of the Tv mushrooms, and have a narrower chemical composition, focused on beta-glucans, spores, attractants to animals that may eat and transport the spores, and protectants to protect the fruitbodies from bacteria and other fungi

The natural ecological role of mushrooms is to assist the breakdown of dead plant matter, and therefore they engage in a highly dynamic interaction with the environment in which they grow. The fungal organism contains a vegetative state consisting of progressive extensions of tissue into a substrate, as well as a reproductive state for spore dispersal (Fig. 1). Mycelium is an aggregation of multinucleate hyphae that typically appear as strands or thin filaments. As a mycelium grows throughout its environment, it secretes an array of compounds into its substrate, altering the chemical nature of the substrate. This enzyme-rich exudate helps catalyze the breakdown of macromolecules for absorption—an example of which are the extracellular lignin-modifying enzymes laccase, lignin peroxidase, and manganese peroxidase [27]. Fungi that engage in this type of enzymatic lignin biodegradation and decompose wood are known as white-rot fungi, named for their white appearance. As a white rot fungus with notable laccase production, Tv has the capacity to enzymatically affect its environment, primarily by degradation of its substrate [28–30]. This enzymatic activity is not limited to lignin-containing woody tissues; rice bran can also function as an efficient substrate for laccase production [31].

Of importance for the understanding of medicinal mushrooms for human consumption, fungal hyphae also secrete a wide variety of defense compounds to deter predators and pathogens [32]. Secreted defense compounds allow the fungi to maintain their territory and evade invasion by bacteria and molds. These compounds may be evolutionarily conserved and offer biological effects for other species such as humans. The medical significance of this is apparent in the famous example of the first antibiotic—penicillin, isolated from the *Penicillium chrysogenum* mold [33].

As structurally different as the mycelium is from the fruitbody, so too are their biological functions. Whereas the mycelium is the major biomass of a fungus and serves to gather nutrients and interact with the substrate during decomposition, the fruitbodies (the most commonly known form of edible mushrooms) are the instruments of spore dispersal in higher fungi (Basidiomycota). They commonly appear as a cap on top of a stem or stalk, with either gills or pore structures underneath the cap. Mycelium and fruiting bodies share similar cell wall structures and contain the polysaccharide complexes that enhance the innate and adaptive immune response [12, 34, 35]. However, concentrations vary, and β-glucans are considered to be present in higher concentrations in the fruiting body compared to the mycelium [36], whereas the mycelial tissue may contain a broader profile of bioactive compounds. Other metabolic variances may exist: recent proteomic research suggests that 40% more protein-coding genes in *G. lucidum* are expressed in the mycelial state, compared to the fruiting body [37].

The production of medicinal mushroom products utilizes a wide spectrum of substrates, including sawdust to mimic the natural habitat, as well as various grains. It is well known that the biological properties of raw grain are altered by fungal fermentation, likely due to the secreted enzymes. A simple fungal organism, namely yeast, grown on red rice, is considered a dietary supplement, and documented to reduce LDL in preclinical and clinical settings [38], properties not associated with consumption of plain unfermented rice. Another example is the *Saccharomyces* yeast-based fermentate EpiCor®, which is composed of the fungal cell walls as well as secreted metabolites produced during the fermentation. The aqueous extract of the dried fermentate has well-documented immune activating, anti-inflammatory, and antioxidant properties [39–41]. Consuming the whole dried fermentate is associated with clinical benefits including improved mucosal immune health [42], and reduced incident and duration of colds [43] and allergies [44]. Research using the SHIME model for digestive health has shown beneficial effects on the gut microbiome [45].

Many medicinal mushroom products are sold as a crude powder consisting of mycelium and its fermented substrate. While pre-clinical and clinical studies have been performed on these products [19, 21], the immunological contributions of the fermented substrate have not been examined. The purpose of this study was to characterize the immunological activity of each of the components, namely the mycelia and the fermented substrate, using the initial substrate as a control. Tv was selected as a model organism for this effort because the physical structure of the mycelium is well defined and allows for harvest of both the mycelium and the fermented substrate (including secreted fungal metabolites) when cultivated in specially designed solid substrate fermentation (SSF) systems. The test model involved evaluation of the early activation marker CD69 on different subsets of immune cells and the induction of production of cytokines and growth factors. The choice of CD69 is based on its role in natural killer (NK) cell function, where CD69 is rapidly induced in NK cells shortly after activation [46] and has a direct role in NK cytotoxicity (killing of target cells) [47].

## Methods

### Reagents

Roswell Park Memorial Institute 1640 medium, penicillin–streptomycin 100×, interleukin-2 (IL-2), phosphate-buffered saline, and lipopolysaccharide (LPS) from *Salmonella enterica* were purchased from Sigma-Aldrich Co. (St Louis, MO, USA). CD69 fluorescein isothiocyanate, CD56 phycoerythrin, CD3 peridinin chlorophyll protein, and heparin Vacutainer tubes were purchased from Becton-Dickinson (Franklin Lakes, NJ, USA). Customized Bio-Plex Pro™ human cytokine arrays were purchased from Bio-Rad Laboratories Inc. (Hercules, CA, USA).

### *Trametes versicolor* (Turkey tail) culture and separation of mycelial and fermented substrate

**Fig. 2:**
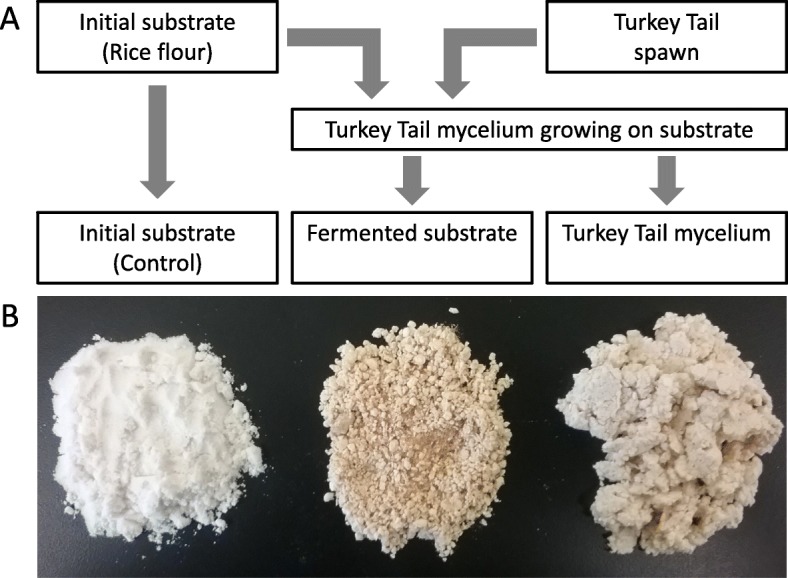
Trametes versicolor (Tv) was used as an experimental model to isolate and compare the mycelium and its fermented substrate. **a** Diagram showing the origin of the three test products compared: Initial substrate (rice flour), fermented substrate, and Tv mycelium. **b** Photo of the three powders: Initial substrate (left) is plain rice flour prior to use as a substrate for growing the Tv mycelium. The fermented substrate (center) is the dried residual powder where the mycelium has been removed. The mycelium (right) is the collection of fungal hyphae, removed from the fermented substrate on which it was grown

The mycelial culture work and sample processing was performed at Fungi Perfecti LLC, following a three-step process of substrate preparation, mycelial culturing, and sample separation (Fig. 2). Certified organic rice flour (Azure Farms, Dufur, Oregon, USA) mixed with water to form a paste and sterilized by autoclaving at 1 bar for 60 min. This resulted in a solid biscuit-like disc of rice grain media (0.4–0.45 g/g water content; aw 0.99). This material constituted the initial substrate (IS). A Petri dish containing 60 g (dry mass) of the milled and sterilized rice flour was inoculated with 50 mg of *Trametes versicolor* agar media spawn. The resulting inoculated media disc solid substrate fermentation microcosm was stored at 20-24 °C for 42 days in a class 1000 clean room. The *Trametes versicolor* mycelium spread radially over the growth substrate, preferentially developing biomass on the surface of the substrate where gas exchange was highest. Mycelium was separated mechanically by removing the surface mycelium from the underlying substrate with a scalpel.

### Preparation of mycelium and substrate for in vitro testing

The three powders were handled in the following manner: 1) Liquid extraction using phosphate-buffered saline (PBS) and referred to as the aqueous fraction; 2) Harvesting the non-aqueous, solid fractions left after aqueous extractions were completed, and passing them through homogenization spin columns (QIAshredder, Qiagen, Hercules, CA). The aqueous fractions were filtered through a 0.22-μm filter before adding to cell cultures. The solid fractions were not filtered through a 0.22-μm filter. This provided two “test products/fractions” from each product, namely the aqueous fraction and the solid fraction. From each fraction, serial dilutions were made in phosphate-buffered saline.

### Dry weight determinations of aqueous extracts

The data graphs show the biological activities per gram starting material. However, in order to understand the relative contributions from aqueous constituents, dry weight assessments were performed for the aqueous fractions. From each of the three powders, a 100 g/L suspension was prepared in distilled H2O. The powder was allowed to hydrate and water-soluble compounds were extracted for 1 h under gentle agitation. Solids were precipitated by centrifugation in conical polypropylene vials for 10 min at 400 g. The liquid fraction was harvested, passed through a 0.22-μm cellulose acetate filter, and dried at 100 °C. The weights of the filtrates were 45 mg/g (4.5%w/w) for the initial substrate, 110 mg/g (11% w/w) for the fermented substrate, and 120 mg/g (12% w/w) for the mycelium.

### Immune cell activation

Peripheral venous blood was drawn from three healthy human donors upon written informed consent, as approval by the Sky Lakes Medical Center Institutional Review Board, Federalwide Assurance 2603. The blood was drawn into heparin vacutainer vials, and the peripheral blood mononuclear cells (PBMC) isolated using Lympholyte Poly (Cedarlane Labs, Burlington, Ontario, CA) by centrifugation for 35 min at 450 g. The PBMC were washed twice in PBS, counted, and the density adjusted to establish cultures with a cell density at 10^6^/mL, using Roswell Park Memorial Institute 1640 medium containing penicillin–streptomycin and 10% heat-inactivated fetal bovine serum (Gibco, Thermo Fisher Scientific, Asheville, NC).

Serial dilutions of products or LPS were added to cultures at a volume of 20 μL, and cultures were then incubated at 37 °C, 5% CO2 for 24 h. The highly inflammatory LPS from *Salmonella enterica* was used as a positive control for immune-cell activation at a dose of 10 ng/mL. In parallel, IL-2 was used as a positive control for natural killer (NK)-cell activation, at a concentration of 100 IU/mL. Untreated negative control cultures consisted of PBMC exposed to phosphate-buffered saline in the absence of test products. All treatments, including each dose of test product and each positive and negative control, were tested in triplicate. After 24 h, blood cells were isolated from each culture well and stained for 10 min with fluorochrome-labeled antibodies at the recommended concentration. PBMC were then fixed using 0.5% formalin. The fluorescence intensities for CD3, CD56, and CD69 were measured by flow cytometry, using an Attune acoustic-focusing flow cytometer (Thermo Fisher Scientific).

During data analysis, gating on forward and side scatter facilitated evaluation of the levels of CD69 expression on lymphocyte and monocyte subsets. The lymphocyte subpopulation was further analyzed for CD69 expression on CD3+ T lymphocytes, CD3+ CD56+ NKT lymphocytes, and CD3- CD56+ NK cells.

### Production of cytokines, chemokines, and growth factors

After 24 h of incubation, the supernatants were harvested from the PBMC cultures described above. Levels of 10 cytokines and chemokines were quantified (IL-1ra, IL-2, IL-4, IL-6, IL-8 (CXCL8), IL-10, interferon gamma (IFN-γ), tumor necrosis factor alpha (TNF-α), MIP-1α (CCL3), and G-CSF) using Bio-Plex Pro™ multiplex immunoassays (Bio-Rad Laboratories, Hercules, CA) and utilizing xMAP technology (Luminex, Austin, TX, USA).

### Statistical analysis

Average and standard deviation for each data set was calculated using Microsoft Excel. Statistical analysis of in vitro data was performed using the 2-tailed, independent *t*-test. Statistical significance was set at *P* < 0.05, and a high level of significance at *P* < 0.01.

## Results

### Induction of the CD69 activation marker on immune cell subsets

**Fig. 3:**
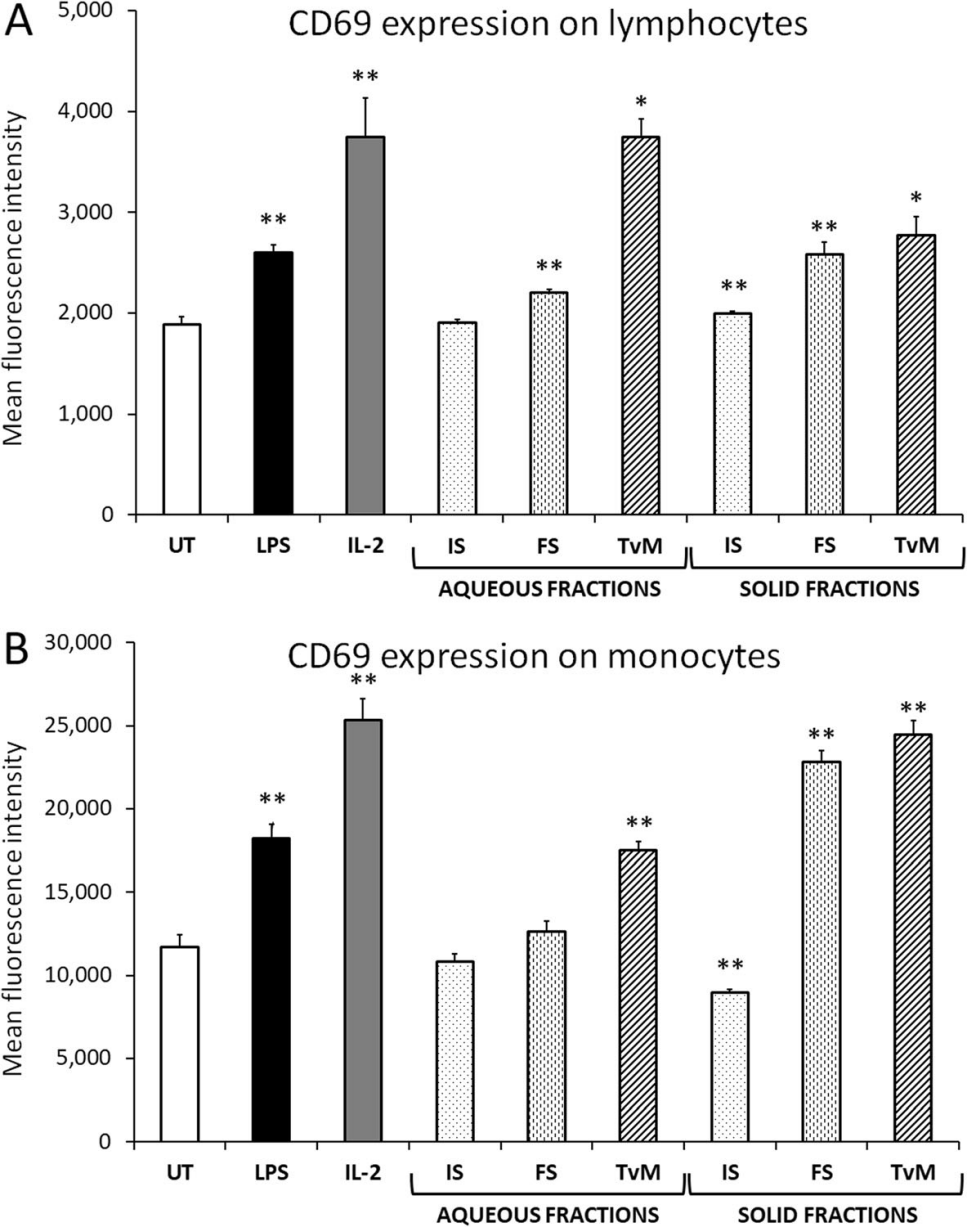
Induction of the CD69 cellular activation marker on lymphocyte (**a**) and monocyte (**b**) subsets in human PBMC cultures. The PBMC cultures were treated for 24 h in the presence of the aqueous versus solid fractions of initial substrate (IS), fermented substrate (FS), and Trametes versicolor mycelium (TvM). Data are shown for the highest dose tested (2 mg/mL), where the dose represents the amount of starting material used to produce a given fraction. Data are presented as mean ± standard deviation of the mean fluorescence intensities in triplicate cultures, and represents one of three separate experiments using PBMC cells from three different healthy human donors. Positive controls included lipopolysaccharide (LPS, 10 ng/mL) and Interleukin-2 (IL-2, 100 IU/mL). Statistical significance is indicated as * for *P* < 0.05 and **for *P* < 0.01

**Fig. 4:**
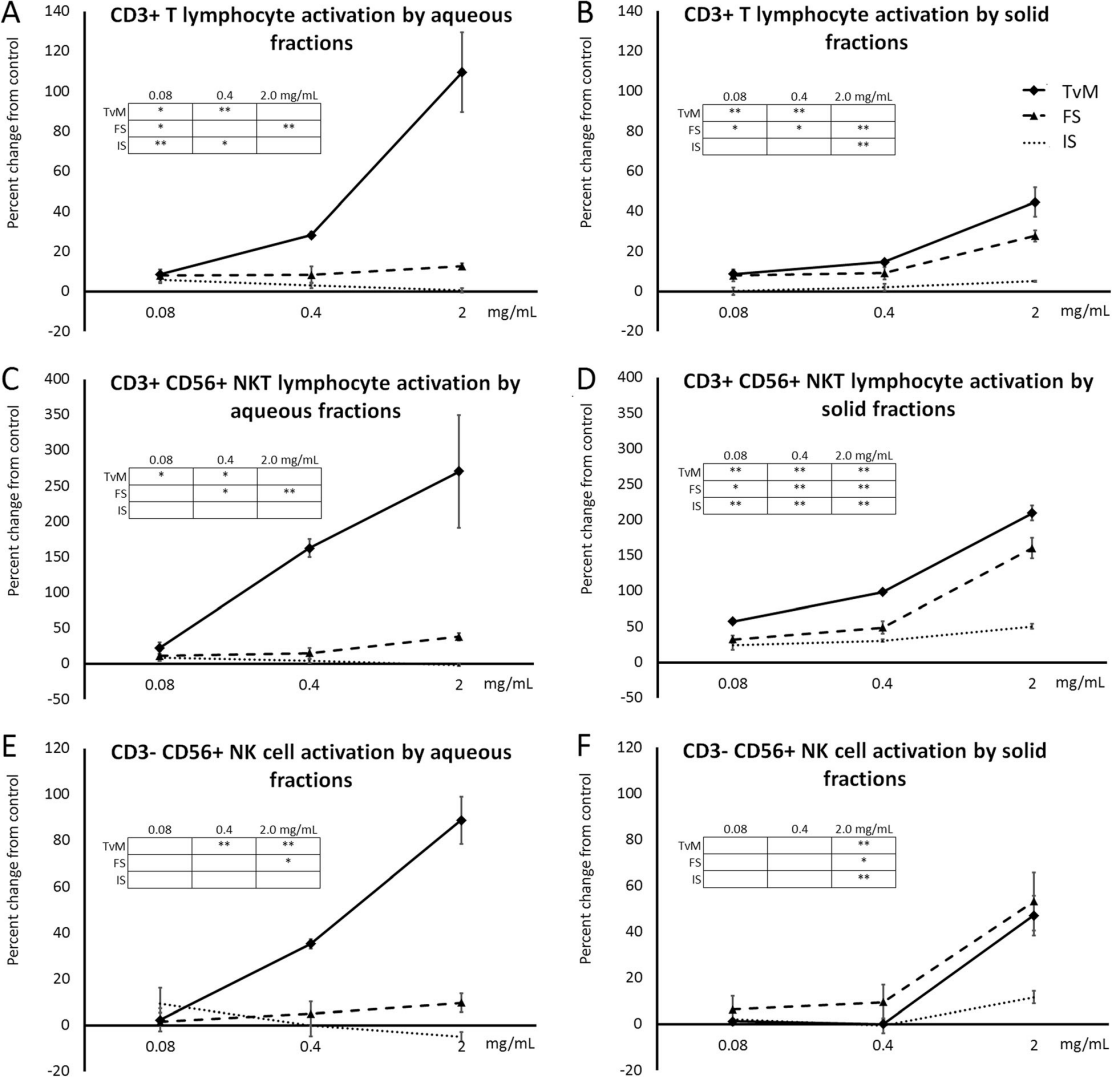
Induction of the CD69 cellular activation marker on immune cell subsets in human PBMC cultures. The PBMC cultures were treated for 24 h in the presence of serial dilutions of Trametes versicolor mycelium (TvM), fermented substrate (FS), or initial substrate (IS). The percent change when compared to untreated control cultures is shown for T lymphocytes (**a**-**b**), NKT cells (**c**-**d**), and NK cells (**e**-**f**). The effects of aqueous extracts are shown in **a**, **c**, and **e**, and the effects of the solid fractions are shown in B, **d**, and **f**. Data are shown for three doses tested (0.08, 0.4, and 2 mg/mL), where the doses represent the amount of starting material used to produce a given fraction. Data are presented as mean ± standard deviation of the percent change seen in triplicate cultures, and represents one of three separate experiments using PBMC cells from three different healthy human donors. Positive controls included LPS and IL-2. The mean ± standard deviation percent change induced by LPS were 19 ± 2.1% for T lymphocytes, 54 ± 8.3% for NKT cells, and 114 ± 10% for NK cells. The mean ± standard deviation percent change induced by IL-2 were 39 ± 0.9% for T lymphocytes, 150 ± 25% for NKT cells, and 446 ± 60.0% for NK cells. Inserted tables: Statistical significance is indicated as * for *P* < 0.05 and ** for *P* < 0.01

The cell surface expression of the early activation marker CD69 was measured on peripheral blood mononuclear cells (PBMC), after 24 h incubation in the absence versus presence of initial substrate (IS), fermented substrate (FS), and *Trametes versicolor* mycelium (TvM). Representative results from one blood donor are shown in Figure 3 and 4, and results from the 2 other blood donors are available in Additional file 1. During flow cytometric data analysis, the gating on the physical characteristics of the cell subsets, allowed analysis on lymphocytes versus monocytes (Fig. 3).

The aqueous fraction of IS showed no effect on CD69 expression (Fig. 3a). The solid fraction showed a very minor increase in CD69 expression on lymphocytes, and a highly significant suppression of CD69 expression on monocytes (*P* < 0.001) (Fig. 3b).

The induction of CD69 on human lymphocytes by the aqueous and solid fractions of FS showed higher CD69 induction by the solid fraction than the induction seen by the aqueous fraction. The difference in CD69 induction by the aqueous and solid fractions of FS was statistically significant (*P* < 0.03).

The treatment of human lymphocytes with both the aqueous and the solid fractions of TvM resulted in a robust and statistically significant increase of the CD69 marker, indicating immune cell activation (Fig. 3a). The induction of CD69 on human lymphocytes by the TvM aqueous fraction was more robust than the induction seen by the TvM solid fraction (Fig. 3a), where the difference between the TvM aqueous and solid fractions was statistically significant (*P* < 0.02).

In contrast, the induction of CD69 on human monocytes by the TvM solid fraction was more robust than the induction seen by the TvM aqueous fraction (Fig. 3b), where the difference between the TvM aqueous and solid fractions was highly significant (*P* < 0.001).

The lymphocyte subset was further analyzed for expression of the CD69 activation marker of CD3+ T cells, CD3+ CD56+ NKT lymphocytes, and CD3- CD56+ Natural Killer (NK) cells (Fig. 4). It was found that the aqueous extract from TvM triggered a very potent activation of NKT cells (Fig. 4c), and a more moderate activation of T cells and NK cells (Fig. 4a and e). The aqueous extract of the fermented substrate only induced minor increases in CD69 on all three cell types, and the aqueous extract of the initial substrate did not induce CD69 on any of the three cell types (Fig. 4a, c, e).

In contrast, the solid fractions of TvM and FS induced comparable levels of cellular activation, as measured by increased CD69 expression (Fig. 4b, d, f). The activation was not as strong as what was seen for the aqueous extract of the mycelium but was stronger than the cell activation by the aqueous extract of the fermented substrate. The solid fraction from the initial substrate showed minor activation of NK cells and NKT cells (Fig. 4d, f).

### Increased production of pro-inflammatory, immune-activating cytokines

**Fig. 5:**
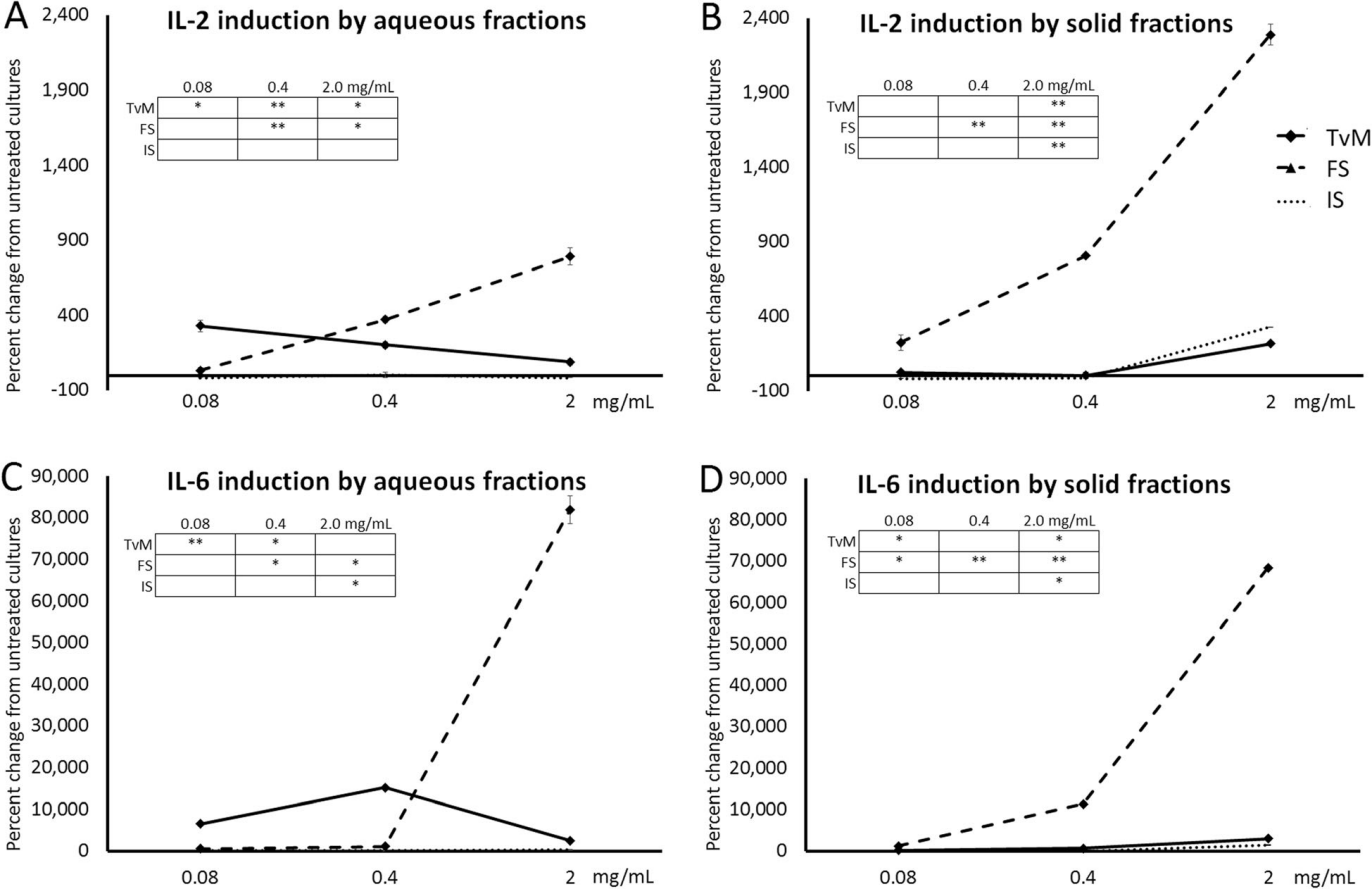
Changes in levels of the cytokines Interleukin-2 (IL-2) and Interleukin-6 (IL-6) in supernatants from human PBMC cultures. The PBMC were cultured for 24 h in the presence of serial dilutions on Trametes versicolor mycelium (TvM), fermented substrate (FS), or initial substrate (IS). The effects on IL-2 and IL-6, cytokines involved in immune activation, of aqueous extracts shown in **a** and **c**, and of the solid fractions are shown in **b** and **d**. Data are shown for three doses (0.08, 0.4, and 2 mg/mL), where the doses represent the amount of starting material used to produce a given fraction. Data are presented as mean ± standard deviation of the percent change seen in triplicate cultures, and represents one of three separate experiments using PBMC cells from three different healthy human donors. Inserted tables: Statistical significance is indicated as * for P < 0.05 and ** for P < 0.01

The culture supernatants from the PBMC cultures were tested for the levels of two cytokines involved in immune cell activation, Interleukin-2 (IL-2) and Interleukin-6 (IL-6). Representative results from one blood donor are shown in Figure 5, and results from the 2 other blood donors are available in Additional file 1. Both the aqueous and the solid fractions of the fermented substrate (FS) induced robust increases in IL-2 and IL-6 levels (Fig. 5). The aqueous fraction of *Trametes versicolor* mycelium (TvM) also induced IL-2 and IL-6, but was more potent at doing so at lower doses (Fig. 5a,c). The solid fraction of TvM had mild effects on IL-2 and IL-6 production in the cultures, and the induction was comparable to the solid fraction of the initial substrate (Fig. 5b,d). The aqueous fraction of the initial substrate did not have any effect on IL-2 or IL-6 induction (Fig. 5a, c).

### Increased anti-viral cytokine production

**Fig. 6:**
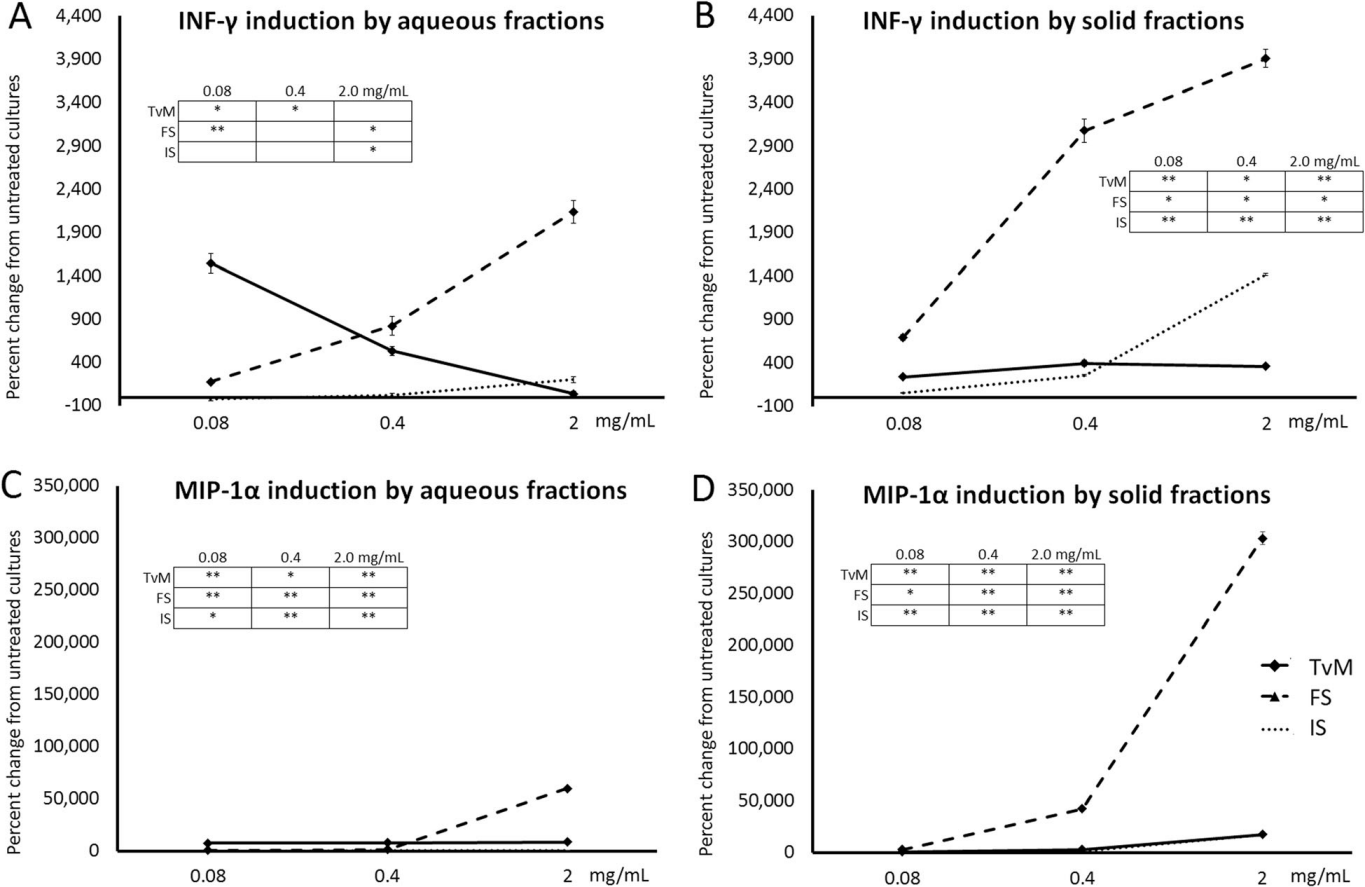
Changes in levels of the cytokines Interferon-gamma (IFN-γ) and Macrophage Inflammatory Protein-1-alpha (MIP-1α) in supernatants from human PBMC cultures. The PBMC were cultured for 24 h in the presence of serial dilutions of Trametes versicolor mycelium (TvM), fermented substrate (FS), or initial substrate (IS). The effects on IFN-γ and MIP-1α, cytokines involved in anti-viral immune defense activity, of aqueous extracts shown in A and C, and of the solid fractions are shown in B and D. Data are shown for three doses (0.08, 0.4, and 2 mg/mL), where the doses represent the amount of starting material used to produce a given fraction. Data are presented as mean ± standard deviation of the percent change seen in triplicate cultures, and represents one of three separate experiments using PBMC cells from three different healthy human donors. Inserted tables: Statistical significance is indicated as * for P < 0.05 and ** for P < 0.01

The treatment of human PBMC with the fungal extracts triggered increased production of two anti-viral cytokines, namely Interferon-gamma (IFN-γ) and MIP-1α (Fig. 6). Representative results from one blood donor are shown in Figure 6, and results from the 2 other blood donors are available in Additional file 1. Both the aqueous and solid fractions from the fermented substrate triggered robust increases in these two cytokines, whereas treatment of cultures with the *Trametes versicolor* mycelium (TvM) led to modest increases in these cytokines. The aqueous fraction of TvM showed more potent effect on IFN-γ at lower doses than at higher doses (Fig. 6a). Interestingly, treatment of human PBMC with the solid fraction of the initial substrate showed a minor increase in IFN-γ and MIP-1α production. The induction of IFN-γ exceeded that induced by the solid fraction of TvM (Fig. 6b). The MIP-1α induced by both aqueous and solid fractions of TvM and the initial substrate were similar in magnitude (Fig. 6c, d).

### Increased anti-inflammatory cytokine production

**Fig. 7:**
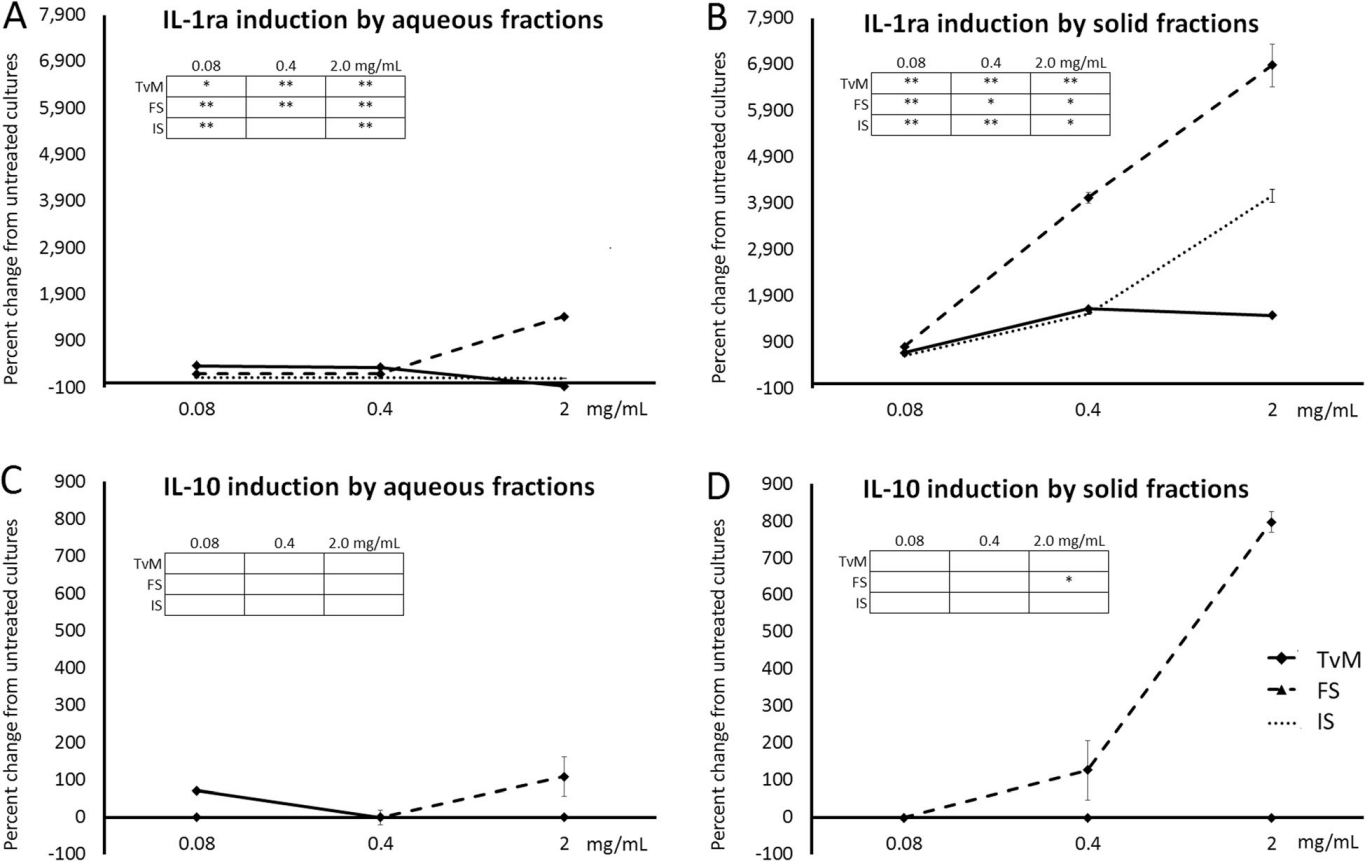
Changes in levels of the cytokines Interleukin-1 receptor antagonist (IL-1ra) and Interleukin-10 in supernatants from human PBMC cultures. The PBMC were cultured for 24 h in the presence of serial dilutions of Trametes versicolor mycelium (TvM), fermented substrate (FS), or initial substrate (IS). The effects on IL-1ra and IL-10, both involved in anti-inflammatory processes as part of the resolution of inflammatory processes, of aqueous extracts are shown in **a** and **c**, and of the solid fractions are shown in **b** and **d**. Data are shown for three doses (0.08, 0.4, and 2 mg/mL), where the doses represent the amount of starting material used to produce a given fraction. Data are presented as mean ± standard deviation of the percent change seen in triplicate cultures, and represents one of three separate experiments using PBMC cells from three different healthy human donors. Inserted tables: Statistical significance is indicated as * for *P* < 0.05 and ** for *P* < 0.01

The treatment of human PBMC with the fungal extracts triggered increased production of two anti-inflammatory cytokines, namely Interleukin-1-Receptor Antagonist (IL-1ra) and Interleukin-10 (IL-10) (Fig. 7). Representative results from one blood donor are shown in Figure 7, and results from the 2 other blood donors are available in Additional file 1. Both the aqueous and solid fractions from the fermented substrate triggered increases in both these two cytokines, with the most robust induction being associated with the solid fraction. The aqueous fraction of *Trametes versicolor* mycelium (TvM) showed more potent effect on both IL-1ra and IL-10 at lower doses than at higher doses (Fig. 7a, c). Interestingly, treatment of human PBMC with the solid fraction of the initial substrate showed a moderate increase in IL-1ra production, exceeding that induced by the solid fraction of TvM (Fig. 7b).

### Increased production of markers involved in regenerative processes

**Fig. 8:**
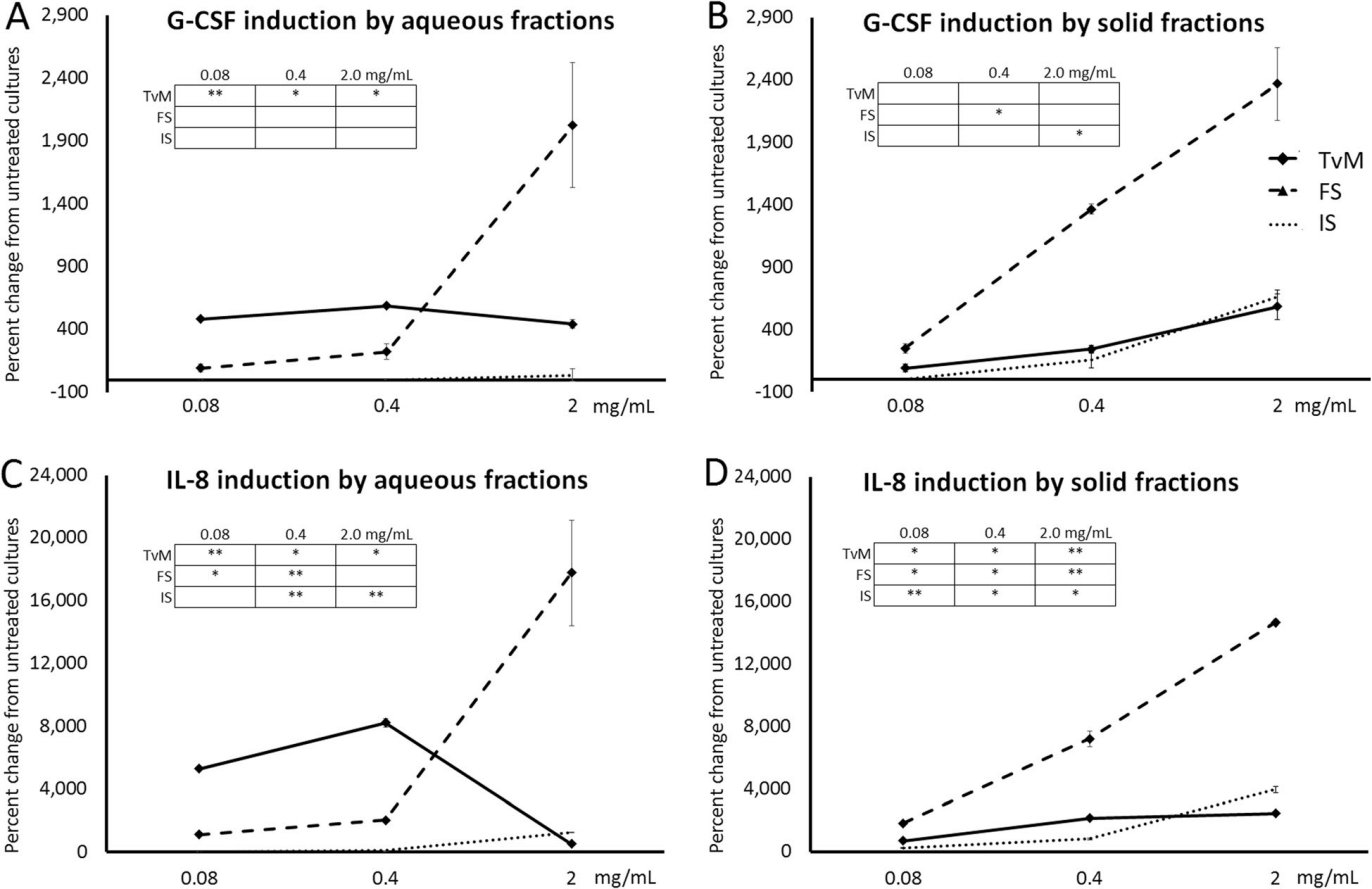
Changes in levels of the growth factor Granulocyte-Colony Stimulating Factor (G-CSF) and the cytokine Interleukin-8 in supernatants from human PBMC cultures. The PBMC were cultured for 24 h in the presence of serial dilutions of Trametes versicolor mycelium (TvM), fermented substrate (FS), or initial substrate (IS). The effects on the stem cell mobilizing growth factor G-CSF and Interleukin-8 (IL-8) of aqueous extracts are shown in **a** and **c**, and of the solid fractions are shown in **b** and **d**.  Data are shown for three doses (0.08, 0.4, and 2 mg/mL), where the doses represent the amount of starting material used to produce a given fraction. Data are presented as mean ± standard deviation of the percent change seen in triplicate cultures and represents one of three separate experiments using PBMC cells from three different healthy human donors. Inserted tables: Statistical significance is indicated as * for *P* < 0.05 and ** for *P* < 0.01

The treatment of human PBMC with the fungal extracts triggered increased production of two biomarkers involved in regenerative processes involving stem cells, Granulocyte Colony-Stimulating Factor (G-CSF) and Interleukin-8 (IL-8) (Fig. 8). Representative results from one blood donor are shown in Figure 8, and results from the 2 other blood donors are available in Additional file 1. For the fermented substrate, both the aqueous and solid fractions triggered increases in both these two markers. The aqueous fraction of *Trametes versicolor* mycelium (TvM) showed effects at a broad dose range (Fig. 8a, c), whereas the effects of the solid fraction from the TvM showed similar effects as the solid fraction from the initial substrate (Fig. 8b, d).

## Discussion

The principal finding of the work reported here was a highly differentiated immune activating effect by the *Trametes versicolor* mycelium (TvM) when compared to its fermented substrate (FM). It was noteworthy that both aqueous and solid fractions of both materials had potent immune modulating activities.

TvM triggered robust increases in the CD69 activation marker on lymphocytes and monocytes, unlike FM which did not induce CD69 on immune cells. The cell surface marker CD69 is rapidly upregulated on many immune cell types after activation, and correlations have been made between Natural Killer (NK) cell CD69 expression and NK cell-mediated tumor-killing activity in the classical target cell-based assay, by a number of research teams over the past 25 years. We have found the induction of the CD69 activation marker a helpful tool for natural products research, both in vitro [48–54] and in clinical studies [55, 56]. When human NK cells are co-cultured with K562 target cells, CD69 expression is upregulated, and the increase significantly correlated with NK cell activity, as measured by today’s gold-standard CD107 mobilization assay [57]. CD69 has the capacity to activate the NK cytolytic machinery in the absence of other NK–target cell adhesion molecule interactions [58]. A direct and highly significant correlation between CD69 levels and NK cell activity was demonstrated by Clausen et al 2003 [59], in a study involving 14 breast cancer patients tested repeatedly during chemotherapy.

NK cells do not function in a vacuum; they are regulatory cells engaged in crosstalk with other cell types [60]. Therefore, the work reported here focused not only on NK cells, but also on NKT cells, T cells, and monocytes. The TvM-mediated induction of CD69 expression on lymphocytes and monocytes was triggered by both aqueous and solid fractions, however, the CD69 expression on lymphocytes was more robust when cells were treated with the aqueous fraction than the solid fraction, with the aqueous fraction comparable to the induction caused by LPS. Given that the aqueous fraction contained almost 10 times less material than the solid fraction, this further demonstrates the potency of the aqueous compounds in the mycelium. For monocytes, this was reversed, where the solid fraction triggered a stronger CD69 expression than the aqueous fraction of the TvM. This is expected, since monocytes are known to be robustly activated by insoluble fungal beta-glucans through Toll-Like receptors (TLR), specifically TLR-2 and TLR-4 [61].

The cytokine induction by aqueous and insoluble compounds in the fermented substrate was seen in the absence of CD69 up-regulation, suggesting activation via alternate pathways, such as has been demonstrated for NK cell activation, which may involve CD69 expression or as an alternative mode of activation involve upregulation of the Interleukin-2 receptor CD25. This was demonstrated by Clausen’s team to be associated with distinct functional differences, where CD69 expression is associated with cytotoxicity as described above, whereas the CD25 expression is associated with increased cell proliferation [59].

Interestingly, the initial un-fermented substrate was not very bioactive, devoid of aqueous bioactive compounds, and only minor effects on cytokine induction by the solid fraction. This further helps demonstrate the uniqueness of the fermented substrate, in terms of fermentation of the rice along with fungal exudates. This is important for the many consumable products that are produced from mycelia along with their fermented substrates.

The aqueous extracts were produced by cold-water extraction, and not using heat or pressure. This is in contrast to other types of fungal extracts and teas, where heat and sometimes pressure is applied to produce the extract. Examples include the use of batch reactors and subcritical water extraction, with heat up to 300 °C, to produce extracts from golden oyster mushrooms (*Pleurotus citrinopileatus*) [62] and Chaga mushrooms (*Inonotus obliquus*) [63].

The results clearly demonstrate that most of the bioactivity for cytokine induction lies in the fermented substrate. Based on the known enzyme secretions by mycelium during active growth, the fermented substrate likely represents a broad array of fungal products, in conjunction with breakdown products from the substrate.

Tv has proved to be an effective model for demonstrating the bioactivities of mycelium versus its fermented substrate. This model will be useful for further evaluation of Tv and other medicinal mushrooms, and this model can help mycology research in the isolation and identification of bioactive compounds. There may be potentially pharmaceutical uses of isolated novel compounds form both mycelium and fermentate, however, one of the more unique and interesting challenges will be to understand their synergistic relationships in traditional medicinal use of the whole crude fermentate. Further work from our team is ongoing and includes this evaluation of synergy, by pretreating isolated NK cells and monocytes with TvM, FM, and a blend thereof, followed by the classical NK activity assay in co-culture with tumor cells, and by co-cultures of Tv-primed monocytes with lymphocytes to evaluate the role of NK cells and monocytes in the overall immunological cross-talk between cell types. In addition, a clinical trial will compare the TvM, FM, and the blend thereof. This will help further document the importance of the multi-faceted and complex actions of the natural mixture of TvM and its fermented substrate, traditionally used for immune support in the integrative medicine setting.

## Conclusions

The work reported here has helped demonstrate that the mycelial fermentation of its substrate dramatically alters the biological effects of the fermented substrate. Furthermore, the mushroom mycelium has distinctly different biological and immune-modulating properties than its fermented substrate. The mycelium was very potent in terms of triggering immune cell activation, whereas the fermented substrate was very active in terms of cytokine induction. Complex immune-activating bioactivity of mycelial-based medicinal mushrooms go beyond effects of insoluble beta-glucans, as potent effects were also seen in the aqueous fraction. The results suggest that overall medicinal effects are associated both with the mycelium itself (including insoluble beta-glucans, but also water-soluble components), and the highly bioactive fermented substrate. Novel applications for animal and human immune health may be identified in the future for components isolated from fermented substrates, independent of mushroom mycelium.

## Supplementary information

**Additional file 1.** The graphs in the paper shows representative data on immune effects on cells from one of three healthy donors. The full sets of data from all 3 blood donors are shown in the supplementary information.

## Data Availability

The datasets used and/or analyzed during the current study are available from the corresponding author on reasonable request.

## Abbreviations

FS: Fermented substrate
G-CSF: Granulocyte-Colony Stimulating Factor
IFN-γ: Interferon-gamma
IL-10: Interleukin-10
IL-1ra: Interleukin-1 Receptor Antagonist
IL-2: Interleukin-2
IL-6: Interleukin-6
IL-8: Interleukin-8
IS: Initial substrate
LPS: lipopolysaccharide
MIP-1α: Macrophage Inflammatory Protein 1-alpha
PBMC: Peripheral Blood Mononuclear Cells
TNF-α: Tumor Necrosis Factor-alpha
Tv: *Trametes versicolor* (Turkey Tail mushroom)
TvM: *Trametes versicolor* mycelium
